# Evolution of cultural traits occurs at similar relative rates in different world regions

**DOI:** 10.1098/rspb.2014.1622

**Published:** 2014-11-22

**Authors:** Thomas E. Currie, Ruth Mace

**Affiliations:** 1 Department of Biosciences, College of Life and Environmental Sciences, University of Exeter, Penryn Campus, Cornwall TR10 9EZ, UK; 2 Department of Anthropology, University College London, 14 Taviton St., London WC1H 0BH, UK

**Keywords:** cultural evolution, cultural phylogenetics, social evolution, phylogenetic comparative methods

## Abstract

A fundamental issue in understanding human diversity is whether or not there are regular patterns and processes involved in cultural change. Theoretical and mathematical models of cultural evolution have been developed and are increasingly being used and assessed in empirical analyses. Here, we test the hypothesis that the rates of change of features of human socio-cultural organization are governed by general rules. One prediction of this hypothesis is that different cultural traits will tend to evolve at similar relative rates in different world regions, despite the unique historical backgrounds of groups inhabiting these regions. We used phylogenetic comparative methods and systematic cross-cultural data to assess how different socio-cultural traits changed in (i) island southeast Asia and the Pacific, and (ii) sub-Saharan Africa. The relative rates of change in these two regions are significantly correlated. Furthermore, cultural traits that are more directly related to external environmental conditions evolve more slowly than traits related to social structures. This is consistent with the idea that a form of purifying selection is acting with greater strength on these more environmentally linked traits. These results suggest that despite contingent historical events and the role of humans as active agents in the historical process, culture does indeed evolve in ways that can be predicted from general principles

## 1. Introduction

Despite being a relatively homogeneous species genetically, humans are characterized by an extraordinary degree of cultural diversity [1,2]. Those concerned with understanding cultural diversity are split between two fundamentally opposed camps. While some argue that there are regular patterns and processes involved in cultural change, others eschew general rules and stress that cultural change and human history is shaped by idiosyncratic and contingent events, and determined by human agency [3–8]. Just as Darwin built up the empirical evidence of biological evolution and the mechanisms responsible for it [9], a key task facing those who argue that there are indeed regularities in cultural change is to build a similar body of evidence that explains the patterns and processes involved in generating the great diversity of human cultures [6,10].

In recent years, a formal theoretical framework has been developed to show how culturally transmitted information may change in ways that are analogous to biological evolution [10]. Mathematical models from population genetics have been adapted to examine how differing modes of transmission of information, e.g. one-to-many, or non-vertical, can affect the evolution of cultural traits [11]. Theoretical and mathematical models of cultural evolution have been well developed for several decades and are now being implemented and assessed in increasing numbers of empirical analyses [12]. For example, recently some of these models have been assessed and tested using laboratory experiments [10,13], field experiments [14] and empirical analyses of cross-cultural [15], linguistic [16], historical [17] and archaeological [18] datasets. Researchers have also applied methods based on phylogenetic trees, developed originally in evolutionary biology, to examine the patterns and processes of cultural evolution at a macro-evolutionary scale [19].

In biology, a key factor in explaining diversity is an understanding of rates of evolutionary change [20,21]. For example, genetic diversity is affected by the degeneracy of the genetic code; at the nucleotide level, certain codon positions will evolve at faster rates because mutations at these sites will be less likely to alter amino acid sequences [22]. Effective population size can influence the rate of evolution, with an increased probability that neutral or even slightly deleterious mutations can drift to fixation in smaller populations [23]. Similar processes may also be invoked to understand variation in cultural systems. Systematic studies of language diversity have recently demonstrated rates of lexical evolution are linked to the frequency with which words are used (suggesting some form of linguistic, purifying selection that leads to slower rates of change in more commonly used words) [24] and the splitting of languages (reflecting either founder effects with smaller population sizes, or the active signalling of identity when new groups emerge) [25]. The evolution of cultural traits that are tested against the environment (e.g. many aspects of technology) is potentially more constrained than other features such as social or ethical norms [26]. Darwinian archaeologists have also argued that patterns of diversity are governed by the differing strengths of selection acting on stylistic and functional features of material culture. While there are only a limited number of ways that arrowheads or pots can be constructed in order to serve their main functional purpose, there is a much greater variety of ways in which the stylistic features, such as decorative designs, can be implemented. Consistent with this idea Rogers & Ehrlich [27] argued that the functional features of Polynesian canoes (e.g. hull construction, presence of outrigger, etc.) show lower rates of change than the stylistic features (e.g. presence of geometric carvings, use of feathers, etc.).

Here, we test the hypothesis that the rates of change of features of human socio-cultural organization are similarly governed by general rules. One possibility raised by this perspective is that different cultural traits will tend to evolve at similar *relative* rates in different world regions, despite the unique historical backgrounds of groups inhabiting these regions. Here, we test whether traits that evolve fastest in one region are also the ones that evolve fastest in another. Although a number of factors might plausibly lead to some traits to change more than others, here we assess whether those traits that are more directly linked to external environmental conditions (e.g. those relating to subsistence and settlements) evolve at a different rate than traits that reflect norms and institutions regulating social relationships (e.g. descent and inheritance systems). ‘Ecological’ traits may evolve more slowly because the most efficient subsistence strategy or the most appropriate building material may be more straightforward to assess and would have direct fitness consequences. This may constrain variation in these ‘ecological’ traits, as inappropriate variants would be less desirable and purifying selection would quickly act to remove them should they be adopted. The success of different ‘social’ traits may be more indirect and harder to evaluate, with the ‘best’ system potentially being very different for different individuals within a society [26,28], resulting in greater change between alternate forms of such traits. Alternatively, if the environment changes relatively rapidly, as may occur during a population expansion into new habitats, then environmental traits may be more liable to change than ‘social’ traits.

To test this hypothesis, we need data that have been coded across a large number of cultures, and some way of being able to track or infer changes over time. While historical or archaeological sources do indeed record changes in human societies, there are currently very few systematic datasets of the required scope or duration [29]. Archaeological information suffers from a secondary limitation in that many features of social organization must be indirectly inferred from the material remains of past societies rather than being directly witnessed. The ethnographic record on the other hand does contain rich information of native forms of social organization based on first-hand descriptions or accounts from informants who lived in such societies. Particularly, relevant for our purposes are systematically coded databases such as the Ethnographic Atlas (EA) [30], which contains information on a range of variables relating to social organization coded into categories based on explicit criteria for a large number of cultures. However, ethnographic data typically lack time depth, making assessments of change problematic.

**Figure 1.**
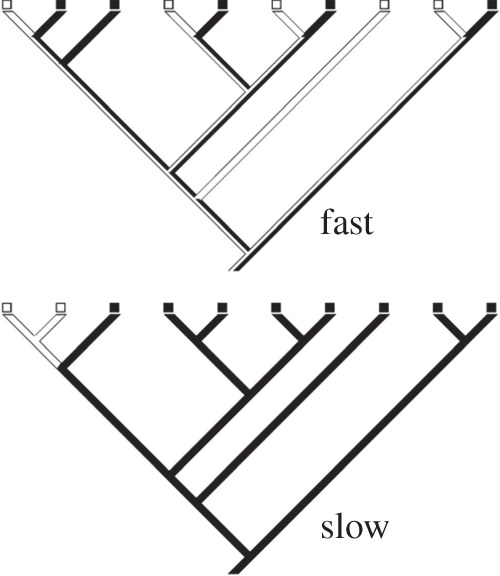
Phylogenetic comparative methods can be used to infer the number of changes in a particular trait that have occurred during the evolutionary history of a collection of ethnolinguistic groups. In this schematic the tree represents the diversification of 10 groups from a single ancestral population. Here a quickly evolving trait MP analysis indicates that the fast trait has changed five times during the evolutionary history of these societies, while the slow trait has changed only once.

Phylogenetic comparative methods provide a solution to these problems [31]. By matching a phylogeny, which represents how different groups are historically related, to ethnographic data, we can make inferences about how different traits have changed over time (figure 1). Previously, phylogenetically informed methods have been used to compare rates of linguistic evolution. Greenhill *et al*. [32] used data from Austronesian and Indo-European languages and found that rates of evolution in typological and lexical features were not substantially different from one another. Dediu [33,34] has also used phylogenetic methods to examine the relative stability of different structural features of language (e.g. linguistic tone and word-order). Importantly, with these methods, we can incorporate different assumptions about the phylogenetic relationships between societies. We can also examine whether results are dependent on the particular method used to infer evolutionary change. For this study, we used linguistic phylogenies and cultural data from two ethnolinguistic groupings: the Bantu-speaking populations of sub-Saharan Africa [35], and the Austronesian-speaking populations of island southeast Asia and the Pacific [36]. These two regions were chosen due to the availability of (i) well-studied language phylogenies constructed using cutting-edge Bayesian techniques and (ii) relatively large numbers of societies (*n* ≥ 100) present with coded ethnographic information in an existing dataset.

## 2. Material and methods

### (a) Ethnographic data

Ethnographic information was taken from Murdock's EA [30]. The EA has a number of advantages for the present purposes; it has a broader coverage of societies than other anthropological databases, such as the standard cross-cultural sample [37], and the data were coded without reference to the particular hypothesis being tested here. Twenty-eight variables, representing a variety of cultural traits, were suitable for these analyses. Some variables required re-coding so that the categories reflected true categories, or to avoid redundancy with other variables. For example, variable 70, *type of slavery*, has a category ‘Reported but type not identified’ that reflects uncertainty in the coding procedure rather a true ethnographic category. In this case, the variable could be re-coded to reflect simply the presence or absence of slavery (see the electronic supplementary material, sections 1 and 4, for a full description).

### (b) Linguistic phylogenies

Following other studies in cultural phylogenetics, we use the inferred relationships between languages to represent the historical connections between ethnolinguistic groups. Large linguistic phylogenies have previously been created using basic vocabulary data for Austronesian [36] and Bantu [35]. One hundred Austronesian languages and 112 Bantu languages from these tree samples could be matched to entries in the ethnographic database (see the electronic supplementary material, section 2). For each variable, the ethnographic data were mapped onto a Bayesian posterior sample of 100 trees from each language grouping. Conducting analyses over these samples means we are not reliant on a single phylogenetic tree but can control for some of the uncertainty about the phylogenetic relationships between the languages.

### (c) Estimating traits changes over phylogenies

Our starting point for assessing the rates of change in different socio-cultural traits is to map the selected ethnographic variables onto the Bantu and Austronesian phylogenies. Using phylogenetic comparative methods, we can assess how many times each trait has changed over both the Bantu and Austronesian phylogenies (see figure 1). Over the same phylogeny, a faster rate of change will generally lead to more trait changes than a slower rate of change. As we are primarily interested in assessing whether the traits that evolve fastest in one region are also the ones that evolve fastest in another (rather than comparing whether traits evolve faster in one region compared with the other) the total number of trait changes is therefore a useful measure (see section *Correlating number of changes across language families* for potential confounds that need to be controlled for when using this measure).

Different comparative methods make different assumptions about the process of trait evolution. Therefore to assess whether our estimates of trait change are robust to such different assumptions here we compare two methods maximum parsimony (MP) [38,39], and the likelihood-based approach of stochastic character mapping (SCM) [40,41] (see the electronic supplementary material, section 6, for a discussion of different methods of estimating numbers and rates of trait change using phylogenetic comparative methods). MP infers the minimum number of evolutionary trait changes that are required to give rise to the observed data given the phylogenetic tree. SCM is a two-step approach that first uses a Markov-chain model of character evolution to infer the instantaneous rate-of-change between different states of the trait using maximum-likelihood, and then uses this rate to simulate changes in the trait over the phylogeny (i.e. probable histories of trait change given the inferred rate of change). MP and SCM therefore use different statistical frameworks but produce output (i.e. inferred number of trait changes) that are directly comparable. For the SCM analyses, 20 simulations (or character maps) were performed for each tree in each posterior sample to capture stochasticity in the inferred number of trait changes. Trait changes for each trait and each tree in each language group were calculated under both methods using the program Mesquite [39]. For each trait, the mean number of changes over the trees (and character maps in the case of SCM) was calculated.

### (d) Correlating number of changes across language families

After calculating the number of changes for each variable in each language family, we used correlations and partial correlations to assess whether the traits that change most in Bantu were also the ones that change most in Austronesian. In order to rule out the possibility of a spurious correlation between the estimated number of trait changes in these two language families, it is important to control for two potential confounding factors. Firstly, when compiling the ethnographic atlas, the authors employed a coding of ‘missing data’ when they felt there was not enough information to make a judgement about what state a particular variable should take for each society. In our analysis, these societies are basically removed from the phylogeny on a trait-by-trait basis and not included in the calculation of the number of trait changes. This means that the effective sample size is different for each variable. For example, the variable *segregation of adolescent boys* could only be coded for 57 of the 112 Bantu societies, while the variable *marriage payments* could be coded for all of them. As a greater number of taxa allows for the possibility of a greater number of changes, a spurious correlation may arise if the sample size varies systematically with the variables across both language families. We therefore use number of societies with coded data for each variable as a control in the correlational analyses.

Secondly, variation in the number of changes in different variables may reflect the number of categories a variable is divided into. For example, the variable *roofing materials* can be coded as one of 10 possible categories, while here the variable *slavery* is coded simply as being present or absent (i.e. two categories). Therefore, partial correlations were conducted between the number of changes in Austronesian and Bantu while controlling for the sample size of each variable in each family, and the number of categories into which the variables are coded. While it could be argued that number of categories itself is reflective of rates of change (a higher rate could lead to more categories being discernible), it could also result from the subjective judgement or expertize of the coder (in this case Murdock). Therefore, in this study in our main analyses, we take the conservative approach that the number of categories needs to be controlled for (see the electronic supplementary material, section 7, to see the effect of including or excluding different control variables).

### (e) Comparing number of changes in ecological and social variables

In order to assess whether ‘ecological’ or ‘social’ variables tend to change more, we classified the variables as falling into one of these two categories. Those traits that relate to direct interactions with the external environment (e.g. subsistence, physical dwellings and settlements), we classified as ‘ecological’, while the remaining variables which relate to norms and institutions of social organization we classified as ‘social’ variables. This classification is indicated in table 1 (see also electronic supplementary material, §3). We compared whether the inferred number of changes under the MP analyses differed between these two classifications within each language family using independent sample *t*-tests. In order to control for the potential confounds mentioned above, we conducted analyses on the unstandardized residuals of linear regressions, with number of changes as the dependent variable, and variable sample size, and number of categories as predictors. In order to assess the magnitude of any significant effect, we use a further linear regression analysis to calculate the familiar *R*^2^ statistic, with these residuals as the dependent variables, and the ecological/social distinction variable as a binary categorical predictor.

**Table 1.** Estimated number of changes in socio-cultural variables. Results are ranked according to increasing residual score in the AN data. Variables classified as ecological (Eco) are marked 1. Cats, number of categories in database; *N*, number of societies for which data are present; Pars, mean parsimony score; SCM, mean stochastic character mapping score; Res, residual of the parsimony score controlling for *N* and Cats*.*

variable information				Bantu				Austronesian			
no.	description	Cats	Eco	*N*	Pars	SCM	Res	*N*	Pars	SCM	Res
83	roofing materials	10	1	109	5.1	5.3	−26.7	77	2.0	2.0	−22.1
42	subsistence economy	3	1	112	4.0	4.1	−12.2	100	10.0	12.3	−15.6
79	dwelling ground plan	6	1	111	12.2	14.2	−10.2	83	6.0	7.0	−14.7
82	shape of roof	9	1	106	26.9	32.4	−2.5	74	10.0	11.9	−11.5
39	animals and plow cultivation	2	1	102	3.0	3.0	−10.6	89	10.1	13.5	−9.9
9	monogamy and polygamy	3	0	111	10.0	11.5	−5.9	95	13.0	16.2	−9.9
29	major crop type	6	1	102	25.3	34.6	2.0	89	17.1	21.4	−9.3
40	predominant type of animal husbandry	7	1	102	20.7	27.3	−5.0	89	20.4	27.3	−7.7
38	segregation of adolescent boys	2	0	57	5.8	7.6	−8.9	71	13.9	24.8	−6.6
27	kin terms for cousins	8	0	74	20.2	27.4	−9.9	95	35.5	56.2	−4.8
30	settlement patterns	8	1	102	36.2	48.9	8.1	89	27.0	37.5	−2.7
34	high Gods	2	0	51	10.0	13.4	−4.0	56	10.9	20.1	−2.2
81	wall material	9	1	60	30.9	60.6	0.1	59	24.1	42.0	0.1
6	marriage payments	7	0	112	13.0	14.1	−12.8	99	31.9	45.3	0.4
32	jurisdictional hierarchy of local community	3	0	102	30.9	52.6	14.9	89	23.8	39.3	2.2
70	slavery	2	0	108	9.9	12.2	−3.1	85	18.5	32.8	2.4
37	male genital mutilations	2	0	90	13.9	19.3	1.0	71	15.1	26.8	2.8
72	succession to the office of local headman	9	0	96	30.5	41.5	0.1	82	31.6	48.2	2.9
15	community marriage organization	6	0	102	47.2	9230.2	23.9	90	33.4	52.2	6.3
33	jurisdictional hierarchy beyond local community	5	0	102	42.4	76.3	21.6	89	33.3	62.8	8.5
80	dwelling floor level	4	1	110	2.0	2.0	−15.3	79	24.0	43.3	8.7
43	descent	7	0	111	28.3	34.9	2.3	100	41.1	70.2	8.8
77	inheritance distribution for movable property	5	0	98	31.8	50.4	12.8	59	17.0	25.3	9.0
66	class stratification	5	0	95	34.8	51.0	13.6	89	36.3	71.8	9.7
24	subtypes of cousin marriages	8	0	86	34.1	56.3	4.7	95	48.8	98.2	11.5
11	transfer of residence at marriage	4	0	112	19.8	24.1	1.3	98	38.0	75.8	11.9
76	inheritance rule for movable property	7	0	98	33.9	48.1	10.0	60	26.9	44.3	15.0
8	domestic organization	8	0	111	39.0	57.2	10.8	97	49.1	112.2	16.9

All statistical analyses involving the estimated number of changes derived from the phylogenetic comparative analyses were conducted using SPSS v. 21.

## 3. Results

### (a) Comparison of estimates from maximum parsimony and stochastic character mapping analyses

MP and SCM produce comparable relative estimates of the number of changes in the cultural traits considered here. The rank order of the mean number of trait changes inferred under MP and SCM is highly correlated in both Austronesian (Spearman's rho = 0.985, *p* < 0.001) and Bantu (rho = 0.972, *p* < 0.001). For the sake of clarity, we focus on the results of the MP analyses. The SCM analyses are described in full in the electronic supplementary material, §7.

### (b) Correlating number of changes

**Figure 2.**
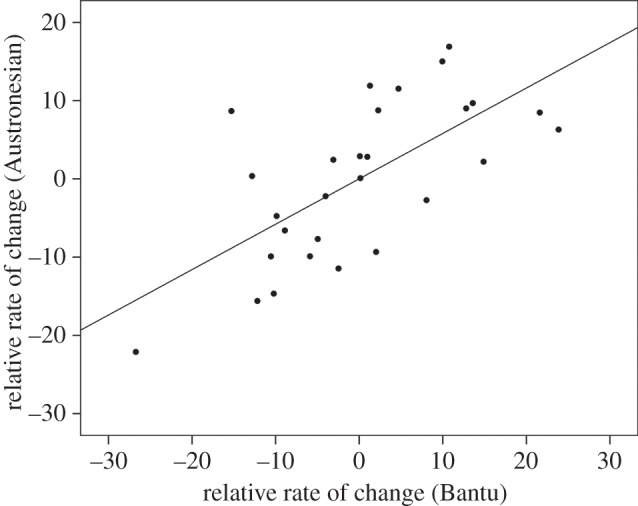
Rate of evolution of cultural traits in Austronesian societies is correlated with rate of evolution in Bantu societies. Rate values in this figure are based on the unstandardized residuals from two linear regressions involving parsimony scores (see Material and methods). Those traits that evolve fastest in Austronesian societies also tend to evolve fastest in Bantu societies. The line is the regression line (*R*^2^ = 0.45).

The number of inferred changes from the phylogenetic comparative analyses for each variable in Austronesian- and Bantu-speaking societies is shown in table 1. The number of changes in these variables in Austronesian and Bantu are significantly correlated in terms of absolute numbers (Pearson's *r* = 0.65, *p* < 0.001) and relative ranks (Spearman's *ρ* = 0.65, *p* < 0.001). This correlation remains even after partialling out the number of categories each variable is coded into, and the number of taxa for each variable in each language family (*r* = 0.65, *p* < 0.001) (figure 2) (the same holds if a non-parametric analysis is performed—see the electronic supplementary material, section 5). The overall patterns are robust to different assumptions about the method of analysis and the variables that need to be controlled for (see the electronic supplementary material, §7). This shows that the similar patterns seen in the relative number of changes in these groups are not merely an artefact of the coding process employed in the creation of the ethnographic database, i.e. more changes are not simply the result of some traits being divided into more categories than other traits.

### (c) Comparing number of changes in ecological and social variables

**Figure 3.**
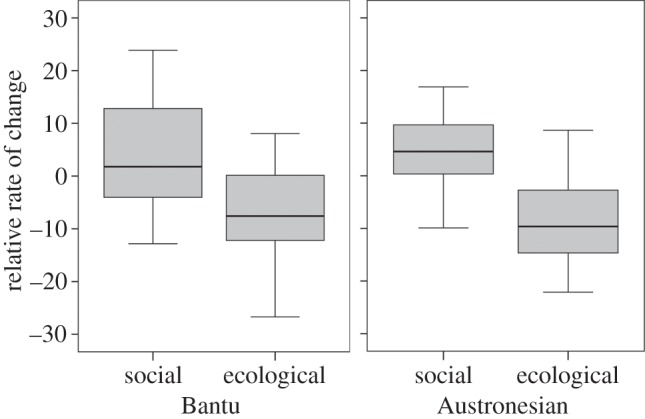
‘Ecological’ variables evolve more slowly than ‘social’ variables. Box plots indicate the distribution of rate values for social and ecological variables in Austronesian and Bantu societies (see Material and methods). Differences in the mean rate values are significant at *p* < 0.05 in both cases.

Ecological variables showed lower amounts of change (controlling for sample size and number of categories) than social variables in both language families (independent sample *t*-test: Austronesian: *t*_26_ = 4.22, *p* < 0.001; Bantu: *t*_26_ = 2.72, *p* = 0.012). (This result holds for non-parametric analyses; see the electronic supplementary material; figure 3 and table 1.) Linear regression with type of variable (social versus ecological) as a predictor returned *R*^2^ values of 0.22 for Bantu and 0.40 for Austronesian. Variables such as *roofing materials*, *subsistence economy* and *dwelling ground plan* showed the least amount of change (controlling for sample size and number of categories) and are all related to external environmental conditions. At the other end of the scale, traits that evolved relatively faster in both groupings were social variables such as *class stratification*, the *inheritance distribution & rule for movable property* and *domestic organization* (see table 1 and the electronic supplementary material, figure S1).

## 4. Discussion

These results indicate that cultural traits in these two cultural regions tend to evolve at similar relative rates, i.e. those traits that change most in Bantu societies are generally the same as those traits that change most in Austronesian societies. This suggests that despite the unique, contingent histories of groups in very different regions of the world similar forces and constraints act on cultural traits making some more labile than others. We investigated whether the degree to which traits are linked to external environmental conditions affects the rate at which cultural traits evolve. Our results support the idea that cultural traits with a more direct environmental basis evolve more slowly than traits related to social and political organization.

It should be emphasized that we are not setting up a false dichotomy between environmental and social dimensions of human societies; the labelling of these traits is to aid comparisons. Our ‘environmental’ traits are of course mediated and perpetuated by social structures, norms and interactions. Equally, the traits we classified as ‘social’ can be plausibly linked to underlying ecological conditions [28,42], albeit at least a step removed from the kinds of environmental traits we have discussed. In other words, while the fitness consequences of employing a sub-optimal subsistence strategy are likely to be severe and immediate, the consequences for a society of having an inefficient inheritance system may be less obvious and may take longer to act on the fitness of individuals. Furthermore, adaptive explanations, such as those consistent with the predictions of behavioural ecology, can still hold for both types of traits [42,43]. For environmental traits, the range of suitable behaviours for a given environment may be relatively limited, thus reducing the probability of change once an optimal solution has been reached. For example, thatching your dwelling may be the best solution for everyone given the availability of materials and technology. However, the evolution of some social variables may be governed more by frequency-dependent processes, and there may be multiple equilibria [2]. The effectiveness of alternative social arrangements may not be much different overall, but the best solution for an individual may depend on what others are doing or on their own circumstances. Interestingly, traits such as *class stratification* and *inheritance distributions* represent situations in which certain individuals may benefit at the expense of others. In societies stratified by hereditary class distinction, those at the lower end of the social scale are vulnerable to oppression or exploitation by the elites. In cases where there are uneven distributions of inheritance, those not in line to inherit may become disaffected. Such structures can lead to social tensions and the relatively rapid evolution witnessed in these traits may reflect the instability of such social arrangements. Even if there are direct or indirect fitness benefits to individuals in such situations [44,45], the long evolutionary history of egalitarianism and preference of equality in our species [46,47] may make such arrangements unappealing and difficult to maintain without the evolution of other norms and institutions that act as cultural ‘work-arounds’ [48].

Our explanation for the fact that the environmental variables evolved at a slower rate assumes that the external environmental influences within these groupings are reasonably stable. Large-scale groupings of societies and languages that are related in a demonstrably phylogenetic manner appear to have arisen from population expansions spurred by the development of agriculture in the Holocene [49]. This primary role for agriculture in these expansions is supported by the predominance of this form of subsistence strategy in both groups and the relatively slow rate of change in this variable in both cultural groupings; it is the second slowest evolving variable in Austronesian societies and the third slowest in Bantu societies. The selection pressures from the external environment acting on historically related groups may be similar for two reasons. Firstly, populations may preferentially expand into environmentally similar regions, as they already possess the technologies suitable for making a living in such an environment [50]. Secondly, rather than just adapting to a new environment, cultures can also modify their environment to suit their existing cultural composition; a form of cultural niche construction [51]. A striking example is the way Austronesian societies modified previously uninhabited islands in the Pacific, bringing with them a ‘transported landscape’ of new plants and animals [52]. An additional point to make is that because of the faster pace at which culture changes (relative to the biological or geological timescales) the external environmental features may also have changed relatively little during the timescale we are considering here: the Austronesian expansion began approximately 5500 years ago [36], while the common ancestor of Bantu groups is thought to have existed 3–5000 years ago [53] (although important changes have no doubt occurred). Interestingly, the rate of one trait, the *dwelling floor level*, was quite discordant between the two language groupings, being relatively fast in Austronesian and relatively slow in Bantu. Many cultures in island southeast Asia, such as the Minangkabau, or the Iban, have houses raised high on stilts, whereas in remote oceanic regions, such as Polynesia, floors were generally formed by the ground or were slightly raised. Several changes between these states appear to have happened with these regions too, and this may reflect important environmental variation relevant to this particular trait within the Austronesian region.

Here, we have only examined the distinction between ecological and social traits as a determinant of the rate of change in cultural traits. As this distinction explains around 20–40% of the residual variation, other factors are also likely to be important in affecting cultural evolutionary rates. The distinction made in studies of material culture between functional stylistic traits may also be applicable to the kinds of socio-cultural features examined in this study. For example, the functional features of rituals that help maintain social cohesion may be relatively constrained, whereas the particular details of these rituals such as the invocation of certain deities, or the particular items of material culture used may be freer to vary. If such stylistic features are used as markers of ethnic identity, then they may show elevated rates of change at certain times, as they are under pressure to change when populations split and establish new cultural groupings [25,54]. Interestingly, previous analyses have demonstrated that the rates of evolution of some linguistic features are very slow, though not through being linked with external environmental conditions. For example, the rate of change of basic vocabulary items has been shown to be linked to the frequency with which they are used [24]. Also the observed slow rate of evolution of linguistic tone may be the result of a genetic bias that favours its acquisition or processing in populations that possess high frequencies of certain genes involved in brain growth and development [33,55]. It is an intriguing possibility that other linguistic or cultural traits could also be affected by such gene-culture coevolutionary processes [6], and this may be discernible through examining their relative rates of evolution.

In this study, we have concentrated on a simple measure of the number of trait changes over a phylogeny as our proxy for the rate of change of cultural traits. This measure is well suited for our present purposes where we are interested in comparing the relative rates of different traits within a language family and assessing whether the traits that tend to evolve fastest (i.e. change more often) in one family also evolve fastest in another. Importantly, our results are robust to controlling for other factors that might affect our ability to link inferred number of changes with relative rate of evolution (i.e. the number of societies for which ethnographic data were available and the number of categories a variable could be coded into). Although not the focus of this study, it is potentially possible to calculate absolute rates of cultural change in terms of number of changes per unit of time. We do not tackle that issue here as the phylogenies we used had branch lengths in units of linguistic change rather than time, and current knowledge about the timing of the Bantu expansion is not that detailed. However, such a measure of absolute rate of cultural change (or other similar measures adapted for quantitative traits or trait change within a population, see [21]) could potentially be used in assessing whether cultural evolution tends to proceed at a faster rate in some regions or in some groups but not others, and could be particularly useful for comparing rates of evolution from other sources of information such as the archaeological or historical records.

The use of phylogenetic comparative methods offers a productive way of testing cultural evolutionary hypotheses. This approach relies on the assumption that the phylogenies used are a good representation of the historical relationships between societies, and the particular cultural traits that are being analysed. However, we know that cultural traits can be borrowed between cultures in a manner analogous to horizontal gene transfer [56], as has been acknowledged and discussed in previous work on cultural phylogenetics (e.g. [19,57,58]). This kind of cultural borrowing can be an important adaptive process, enabling beneficial traits to spread (e.g. the horse in the North American Plains [59] (cited in [60]), writing systems across Eurasia [50]). From a theoretical perspective for our present purposes of understanding rates of evolution, it does not matter particularly whether trait changes are due to independent change, convergent evolution or borrowing. While from a practical perspective, previous work using computer simulations has demonstrated that accurate inferences involving detecting correlated evolution between traits are possible even when borrowing does occur [57]. Furthermore, Dediu & Levinson [34] argue that the stability of linguistic features as estimated by their phylogenetic method accords well with other linguistic studies that have incorporated horizontal processes. Potentially, borrowing could have the effect of making closely related cultures more similar to each other, in which case we might underestimate the rate of change as a widely borrowed trait may incorrectly be inferred to have arisen at an earlier point. However, borrowing, particularly between less closely related groups, could also lead to patterns that lead us to increase our estimate of the rate of change. Such processes may indeed cause problems in trying to accurately reconstruct the cultural traits of a particular society in the past, but that is not our task here. The precise impact that borrowing will have on the estimates from phylogenetic comparative methods will ultimately depend on where and when it occurs and what form it takes [57]. A small number of horizontal transfers is unlikely to have dramatically affected our estimates of the parameters of interest over all the traits examined here [34,57], and it seems unlikely that this could have introduced a large systematic bias that would be necessary to lead to the observed correlation between the number of changes in Austronesian and Bantu societies.

Overall, these results suggest that despite contingent historical events and the role of humans as active agents in the historical process, culture does indeed evolve in ways that can be predicted from general principles [6,10]. While some formulations of cultural evolution have been mainly metaphorical and relied on verbal arguments [61], a formal body of cultural evolutionary theory is now well established, the assumptions and predictions of which are being tested by an increasing number of empirical studies from a variety of disciplines. Indeed, evolutionary theory can act as a unifying force in the social sciences to help the insights from multiple disciplines be more readily shared and synthesized [8,10]. A more systematic understanding of how culture evolves is not only of academic interest but also can help in better understanding the ways in which new ideas arise and spread, and the most effective ways of changing norms and institutions to help solve some of our most pressing social problems [26,62,63].

## Data accessibility

Data available from the Dryad Digital Repository: http://doi.org/10.5061/dryad.pv84f.

## Supplementary Material

ESM
