# Supplementary material for: Evolution of cultural traits occurs at similar relative rates in different world regions
Source: Proc Biol Sci. 2014 Nov 22;281(1795):20141622. doi: 10.1098/rspb.2014.1622 (PMC4213619; doi:10.1098/rspb.2014.1622)
Supplement: ESM [file rspb20141622supp1.pdf]

Cultural Traits Evolve at Similar Relative Rates in Different World Regions

Thomas E. Currie & Ruth Mace

ELECTRONIC SUPPLEMENTARY MATERIAL

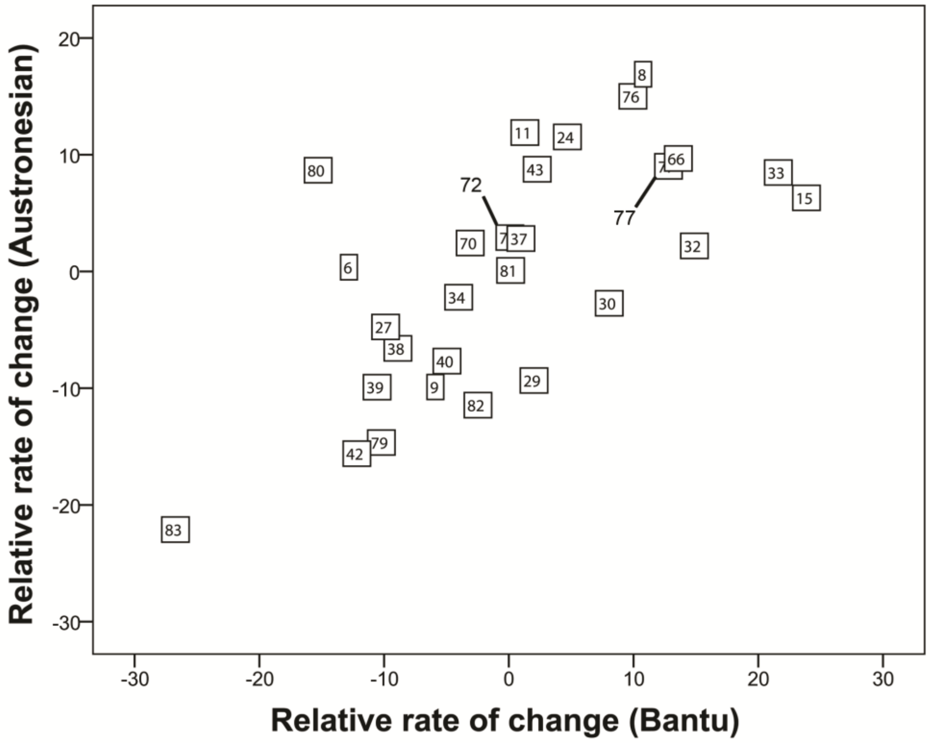

Fig S1. *Rate of evolution of cultural traits in Austronesian societies is correlated with rate of evolution in Bantu societies. Redrawing of figure 2 with data points labelled with variable numbers from Table 1.*

1. Coding of ethnographic data

Not all variables in the Ethnographic Atlas were suitable for this analysis. Reasons for not including variables were: 1) variables on Sex Differences and Task Specialization rely on the practice being present to be meaningful, 2) variables which are overtly continuously distributed (e.g. variable 31, *mean size of local communities*) would result in arbitrary categorization, 3) redundancy between variables (e.g. variable 43, *Descent*, summarizes three other variables, which are therefore not included individually), 4) characters are invariable in

at least one of the language families (meaning the rate of change is not measurable), 5) variable relates to a secondary rather than a primary form.

Where possible the coding of ethnographic variables into categories that was employed by Murdock was retained. However, some variables were re-coded into a smaller number of categories to enable phylogenetic comparative analyses. Variable 9, *Marital Composition: Monogamy and Polygamy*, was condensed from 7 categories to 3: Monogamy (categories 1 and 2), Polygamy (categories 3-6), and Polyandry (category 7). This was done in order to more fully distinguish variable 9 from variable 8 (Domestic organization). Variable 34, High Gods, was re-categorized into two categories reflecting simple presence versus absence, rather than more fine-grained subjective distinctions about the role of such gods within a society. Variable 37, *Male Genital Mutilations*, had categories based on rather arbitrary age classifications (10-20, 30-40 etc.) at which such mutilations occur. Therefore this variable was re-categorized to reflect the presence or absence of male genital mutilations (present: categories 2-10; absent: category 1). Similarly variable 38, *Segregation of Adolescent Boys*, was re-categorized into presence versus absence (present: categories 2-5; absent: category 1), as there were several categories reflecting different levels of segregation. Variable 39, *Animals and Plow Cultivation*, was also recategorized into presence versus absence (present: categories 2 and 3; absent: category 1), as the present categories only distinguish between the use of plow being aboriginal prior to contact or not. For variable 42, Subsistence economy, the category "agriculture, type unknown", means that the most sensible categorization for the present purposes was to have three categories (Foraging: categories 1-3; Pastoralism: category 4; Agriculture: categories 5-9). Variable 70, *Type of Slavery*, has a category "reported, but type not identified" meaning that it had to be recategorized as presence/absence (present: categories 2-4; absent: category 1). A copy of Murdock's ethnographic data for the Austronesian and Bantu societies listed in Section 2 can be found at doi:10.5061/dryad.pv84f, with variable coding adjusted in the manner outlined above.

2. Matching Ethnographic Variables to Languages

Bantu Language	Ethnographic Atlas	Austronesian Language	Ethnographic Atlas
Ejagham (800)	EKOI	AklanonBisayan	BISAYAN
Tiv (802)	TIV	Bajo	TAWI-TAWI
Ngyemboong d.bangang (951)	BAMILEKE	Bali	BALINESE
Duala (A24_2)	DUALA	Bima	SUMBAWANE
Bubi (A31)	BUBI	BontokGuinaang	BONTOK
Puku (A32_1)	PUKU	Bunun	BUNUN
Bakoko (A43b)	KOKO	Bwaidoga	BWAIDOGA

Rikpa (1_A53_1)	BAFIA	Canala	AJIE
Fang (A75_1)	FANG	Cebuano	SUGBUHANO
Mpongwe (B11a)	MPONGWE	CentralAmis	AMI
Kota (B25_1)	KOTA	Chamorro	CHAMORRO
Tsogo (B31)	SHOGO	Chru	RHADE
Teke (B73_5)	TEKE	ChuukeseAKATrukese	TRUKESE
Madzing (B80_E)	DZING	Dehu	LIFU
Yanz 1 Kibongo (B85_1)	YANZI	Dobuan	DOBUANS
Sakata (C34)	SAKATA	ElatKeiBesar	KEI
Lingala (C36)	NGALA	FijianBau	MBAUFIJIA
Buja 1 Monongo (C37_1)	BUDJA	FutunaEast	FUTUNANS
Doko (C40_1)	NDOKO	GhariGuadalcanal	KAOKA
Ngombe (C41)	NGOMBE	Hanunoo	HANUNOO
Mbesa (C51)	BOMBESA	Hawaiian	HAWAIIANS
Poke 1 d. Lyombo (C53_1_)	TOPOKE	HituAmbon	AMBONESE
Likile (C57)	LOKELE	Iban	IBAN
Mongo (Nkundo_C61_1)	NKUNDO	Idaan	DUSUN
Mongo (C61_3)	MONGO	IfugaoBatad	IFUGAO
Mongo 4 d. Konda (C61_4)	EKONDA	Javanese	JAVANESE
Tetela (C71_1)	TETELA	KalingaGuinaangLubuagan_D	KALINGA
Kucu (1_C72_1)	KUTSHU	Kambera	SUMBANESE
Kela (C75)	KELA	KankanayNorthern	SAGADA
Lele (C84_1)	LELE	Kapingamarangi	KAPINGAMA
Songe (D10S)	SONGE	Kilivila	TROBRIAND
Binja (D24_1)	SONGOLA	Kiribati	ONOTOA
Lega (D25_3)	REGA	Kodi	KODI
Bira (D32_2)	BIRA	KoronadalBlaan	BILAAN
Bodo 1 Badimbia (D35_1)	BUDU	Kusaie	KUSAIANS
Kumu (D37_2)	KUMU	Kwaio	KWAIO
Gikuyu (E51)	KIKUYU	Lakalai	LAKALAI
Kamba (E55)	KAMBA	Lamaholot	ILI-MANDI
Caga (E62_1)	CHAGGA	Lau	LAU
Pokomo (E71_1)	POKOMO	Levei	MANUS
Giryama (E72a_1)	GIRIAMA	Luangiua	ONTONG-JA
Digo (E73_1)	DIGO	Makassar	MACASSARE
Teita (Sagala_E74_B)	TEITA	MaloSantaCruz	SANTACRUZ
Sukuma (F21)	SUKUMA	Manam	MANAM
Nyamwezi (F22)	NYAMWEZI	Mangareva	MANGAREVA
Sumbwa (F23)	SUMBWA	Maori	MAORI
Gogo (G11)	GOGO	Marquesan	MARQUESAN
Kaguru (G12)	KAGURU	Marshallese	MARSHALLE

Pare 1 Malindi (G22_1)	PARE	Mekeo	MEKEO
Shambala (G23)	SHAMBALA	Melayu	MALAYS
Zigula (G31)	ZIGULA	MerinaMalagasy	MERINA
Ngwele (G32)	KWERE	Minangkabau	MINANGKAB
Ngulu (G34)	NGULU	Moken	SELUNG
Luguru (G35)	LUGURU	Molima	MOLIMA
Hadimu (G43_C)	HADIMU	Mortlockese	NOMOIANS
Kinga (G65)	BENA	Mota	MOTA
Yombe (H12b_1)	YOMBE	Motu	MOTU
Sikongo (H16h)	KONGO	Nahavaq	SENIANG
Sundi (H16i_4)	SUNDI	Niue	NIUEANS
Yaka (Kasongo_H31_2)	SONGO	Paiwan	PAIWAN
Yaka (H31_4)	YAKA	Palauan	PALAUANS
Suku (H32)	SUKU	Penrhyn	TONGAREVA
Hima (J13)	NYANKOLE	PhanRangChamEasternCham	CHAM
Ganda (J15)	GANDA	Ponapean	PONAPEANS
Soga (J16)	SOGA	PunanKelai	PUNAN
Regi (J20)	KARA	Puyuma	PUYUMA
Haya (J22_H)	HAYA	RapanuiEasterIsland	EASTER
Zinza (J23)	ZINZA	Rarotongan	MANGAIANS
Kerebe (J24)	KEREWE	Rennellese	RENNELL
Hunde (J51)	HUNDE	RotiTermanu_D	ROTINESE
Shi (J53)	BASHI	Rotuman	ROTUMANS
Rwanda (J61_1)	RUANDA	Saa	ULAWANS
Rundi (J62)	RUNDI	SaipanCarolinian	CAROLINIA
Ciokwe (K11)	CHOKWE	Samoan	SAMOANS
Lwena (K14_1)	LUVALE	Selaru	TANIMBARE
Gangela (K19)	LUIMBE	Simbo	SIMBOESE
Lunda (Ndembu_K22_1)	NDEMBU	SquliqAtayal	ATAYAL
Mbala 1 Ivukuna (K51_1)	MBALA	Suau	DAHUNI
Pheende (K52)	PENDE	SubanunSindangan	SUBANUN
Luba-Ks (L31)	LULUA	SyeErromangan	EROMANGAN
Luba (L33)	LUBA	TaeSToraja	TORADJA
Sanga (L35)	SANGA	TagbanwaAborlan_D	TAGBANUA
Kaonde (L42)	KAONDE	TahitianModern	TAHITIANS
Mambwe (M15)	MAMBWE	TannaSouthwest	TANNESE
Nyakyusa (M31_2)	NYAKYUSA	Teop	KURTATCHI
Bemba (M42)	BEMBA	Tetum	BELU
Lala (M52)	LALA	Tikopia	TIKOPIA
Lamba (M54)	LAMBA	TobaBatak	BATAK
Tonga (M64_1)	PLTONGA	Tokelau	TOKELAU
Tumbuka (N21_2)	TUMBUKA	Tongan	TONGANS
Nyasa (N31)	NYASA	Tuamotu	RAROIANS
Nyanja (N31a)	NYANJA	Tuvalu	ELLICE

Cewa (N31b)	CHEWA	UveaEast	UVEANS
Kunda (N42)	KUNDA	VaghuaChoiseul	CHOISEULE
Sena (N44)	SENA	Waropen	WAROPEN
Yao (P21_2)	YAO	WBukidnonManobo	MANOBO
Makwa (P31_1)	MAKUA	Wogeo	WOGEO
Umbundu (R11_1)	MBUNDU	Woleaian	IFALUK
Ndonga (R22)	AMBO	Wuvulu	AUA
Herero (R31)	HERERO	Yami	YAMI
Shona (S10)	SHONA		
Ndau (S15)	NDAU		
Venda (S21)	VENDA		
Tswana (S31)	TSWANA		
Sotho (S33)	SOTHO		
Lozi (S34)	LOZI		
Xhosa (S41)	XHOSA		
Zulu (S42)	ZULU		
Swati (S43)	SWAZI		
Ndebele (S44)	NDEBELE		
Ngoni (S45)	NGONI		
Tsonga (S53)	THONGA		

Table S1. *Names of Bantu languages (with Language Codes in parentheses, following Bastin [1]), and names of Austronesian languages (from the ABVD [2]) matched to ethonyms in the Ethnographic Atlas.*

3. Ecological versus Social variables

For the purposes of the present study a judgment was made by the authors about whether variables should be classified as "ecological" (i.e. relating to direct interactions with the external environment, such as subsistence, physical dwelling, and settlement variables), or "social" (i.e. relating to norms and institutions of social organization). Again we stress that this distinction is not designed to suggest that social traits are completely divorced from external environmental conditions. Ideally, for future research it would be desirable to have some objective measure of the degree to which such variables relate to external environmental conditions.

Interestingly, in a previous study Guglielmino and colleagues [3] argue that traits from the Ethnographic Atlas that broadly match our "ecological" variables showed the strongest associations with a measure of ecological similarity in terms of habitat type, while traits relating to kinship and social organization are less strongly associated, with all traits showing some degree of association with linguistic similarity. This is broadly consistent with our argument in this study, however it should be noted that this earlier study has a number of methodological short-comings including rather course-grain measures of linguistic and ecological similarity (the latter of which may be more or less suitable for different traits), and the non-independence of the units of analysis [4]. Furthermore, this previous study was

concerned with addressing whether language, ecology, or geographic distance (as a proxy for borrowing) was the best predictor of cultural trait diversity in Africa, which is a somewhat different question to the present study (see section 8).

4. Inclusion of inheritance variables

The EA contains two variables that relate to inheritance rules (i.e. matrilineal, patrilineal, etc. variable 74, *Inheritance Rule for Real Property (Land)*, and variable 76, *Inheritance Rule for Movable Property*), and two variables that relate to the distribution of inheritance (i.e. all eligible offspring inherit equally, primogeniture etc., variable 75, *Inheritance Distribution for Real Property (Land)*, and variable 77, *Inheritance Distribution for Movable Property*). To assess whether these variables for land and movable property are redundant with each other the correlation between them was assessed through visual inspection of contingency tables. These tables show that cultures tend to be categorized similarly for both the inheritance rule (Table S2) and inheritance distribution (Table S3) variables (i.e. cultures that practice primogeniture for real property, also tend practice primogeniture for movable property. It was therefore decided that only one inheritance rule variable, and one inheritance distribution variable should be used. The variables relating to movable property were therefore chosen as they have data for a slightly higher number of taxa in both AN and BN.

		v76							Total
		1	2	3	4	5	6	7	
v74	1	1	1	1	0	2	0	0	5
	2	1	5	0	0	0	0	0	6
	3	1	0	2	0	0	0	1	4
	4	0	0	0	2	1	0	0	3
	5	0	0	0	0	17	0	0	17
	6	0	0	0	0	0	1	0	1
	7	0	0	0	0	1	0	18	19
Total		3	6	3	2	21	1	19	55

Table S2. *Comparison of frequency distributions of variable 74 (v74, Inheritance Rule for Real Property (Land)), and variable 76 (v76, Inheritance Rule for Movable Property) in Austronesian societies. Societies tend to be classified into similar categories in both variables, indicating both variables are redundantly tapping into the underlying variable relating to inheritance rules in general.*

			v77				Total
		1	2	3	4	9	
v75	1	28	0	0	0	2	30
	2	1	0	0	0	0	1
	3	0	0	2	0	0	2
	4	1	0	0	9	0	10
	9	4	0	0	0	1	5
Total		34	0	2	9	3	48

Table S3. *Comparison of frequency distributions of variable 75 (Inheritance Distribution for Real Property (Land)), and variable 77 (v77, Inheritance Distribution for Movable Property) in Austronesian societies. Societies tend to be classified into similar categories in both variables, indicating both variables are redundantly tapping into the underlying variable relating to inheritance distributions in general.*

5. Non-parametric analyses

There is a moderately strong and significant correlation between the rank order of the estimated number of changes in Austronesian and Bantu societies under Maximum Parsimony (Spearman's rho_{28}=.65, p<0.001). To control for the potential confounds in a manner similar to partial correlation two linear regression analyses were performed with the number of AN societies for each trait, the number of BN societies for each trait, and the number of Ethnographic Atlas categories as predictors of the number of changes in AN and BN. The rank correlation of the unstandardized residuals from these analyses was then calculated. There is a moderately strong and significant correlation between the rank order of the residuals (rho_{28}=.651, p<0.001), indicating that the rates of change in these two groups of societies follow approximately the same order (i.e those traits that evolve fastest in AN, also evolve fastest in BN)

Mann-Whitney U tests (non-parametric equivalent of the t-test) show that the residual scores of the "Social" variables are ranked significantly higher than the residual scores of the "Ecological" variables in both language families (Bantu, U_{26}=40, Z=-2.40, p=0.017; Austronesian U_{26}=20, Z=-3.356, p=0.001).

6. Alternative ways of modelling trait evolution in phylogenetic comparative methods

Phylogenetic comparative methods cover a wide range of different statistical approaches to tracing the evolutionary history of traits over a phylogenetic tree. These different approaches provide flexibility and the ability to address a wide range of evolutionary hypotheses, for both continuously- and discretely-distributed characters. They can often involve very different assumptions about how evolutionary change is modelled, therefore it can be important to assess whether different methods or statistical approaches produce the same results. Broadly speaking for discrete traits (such as those used in this study) these methods can be divided into those approaches that either attempt to trace character changes with reference to some kind of optimality criterion, or propose a statistical model of evolutionary change and infer the parameters of this model [5].

In this study we use both approaches in employing two methods: 1) Maximum Parsimony (MP), and 2) Stochastic Character Mapping (SCM).

Under MP we estimate the minimum number of trait changes required to produce the observed distribution of trait states over the phylogenetic tree. The advantage of this approach is that it is intuitive and produces output (i.e. the number of inferred trait changes) that is straightforward to interpret for our purposes. Another practical consideration is that is computationally efficient and produces results relatively quickly (an important point when the number of traits to be analysed is large, as is the case in this study). Legitimate criticism of parsimony is that some of its assumptions are not explicit, i.e. it does not employ an explicit model of character evolution, and implicitly assumes that the rate of evolution is relatively slow with respect to the phylogeny. Another criticism is that it does not make use

of the information contained in the branch lengths of the phylogenetic tree (one feature of this method is that only a single change can occur along each branch of the tree).

SCM was chosen as a model-based approach that produces output (i.e. inferred number of changes) that is directly comparable to that of MP. SCM (as implemented in the Mesquite program) infers the rate parameters of a statistical model of trait evolution using Maximum Likelihood (ML). It then uses this information to propose possible character changes over the phylogeny (i.e. trait histories, or character maps) that are consistent with this rate of evolution and the probabilities of different trait states at internal nodes in the phylogeny. It allows multiple changes along the branch lengths of the phylogeny and makes use of information about branch lengths such that more changes are likely along longer branches. Thus the estimated number of changes under SCM will be equal or greater than those under MP.

SCM (and other approaches such as the DISCRETE, and MULTISTATE algorithms in BayesTraits, *ace* command in the R package *ape*) are based on a Markov-chain model of character change, where traits are modelled as switching between different states over an infinitesimally small interval of time. The parameters of these models are these *instantaneous* transition rates between different states, and are calculated with reference to the branch lengths of the phylogenetic tree (which are in units of linguistic change rather than time), i.e. they are not directly interpretable as number of changes, or changes per unit of time. Different models of evolution are possible by specifying whether these rate parameters should be estimated separately, whether certain parameters should be set to be equal to each other, or whether some parameters should take a value of zero (meaning that certain changes from one state to another cannot occur). For traits that take more than two states the Mesquite program used here only allows a model of evolution with a single rate parameter for all possible transitions, i.e. a trait can change from one state to any other at the same rate. This model is obviously an oversimplification, and interesting evolutionary hypotheses can be tested by examining competing models of trait evolution (e.g. [6, 7]). However, there may not always be enough information in the data to justify fitting a more complex model with differing rate parameters [8]. The number of possible models of evolution increases dramatically as the number of states a trait can take increases [9]. Ideally, assessing the appropriate model of evolution should be done with care and with reference to relevant existing theory and other lines of data. Such a task is beyond the scope of this study given that the particular model of evolution is not our focus *per se*, and the fact that we are examining a large number of traits. Even if models with more than one rate parameter were to be estimated it is unclear how these values could be combined meaningfully to produce an overall rate of change for the trait. Given these complexities associated with interpreting these instantaneous rate parameters here we have chosen to focus on the estimated number of trait changes produced by SCM under the single rate model of evolution. Given that we control for the number of states a trait can take, there is no reason to suspect that this measure leads to the observed correlation between relative rates of change in Bantu and Austronesian

even if it is ultimately based on a rather simplified model of evolution. For completeness, we include the instantaneous rate parameter values under ML as a further point of comparison. As the next section demonstrates, the inferences made under these different comparative methods are broadly comparable; meaning our finding that the relative rates of change in these traits are correlated across the two language families is robust to different assumptions by different methods.

7. Comparison of inferred number and rates of change under MP, SCM, and ML analyses

As figure S1 indicates both MP and SCM make comparable inferences about the relative number of changes in both Austronesian and Bantu. The figure also confirms that SCM estimates are always equal to, or higher than estimates under MP. Spearman's rank correlation analyses show that these estimates are highly correlated (Austronesian: rho=0.985, $p<0.001$; Bantu: rho=0.972, $p<0.001$). The non-parametric correlation is given here because in Bantu variable 15 (*Community Marriage Organization*) SCM analyses produced an extremely high number of mean estimated changes due to a high estimated instantaneous rate of change. The mean number of changes estimated was more than 9000, which is around two orders of magnitude greater than the other traits, and is a substantial outlier. If this variable is not included the mean number of trait changes inferred under MP and SCM is highly correlated in both Austronesian (Pearson's $r = 0.971$, $p<0.001$) and Bantu ($r=0.972$, $p<0.001$). In subsequent results using SCM or ML results, this variable has been omitted due to its status as a substantial outlier.

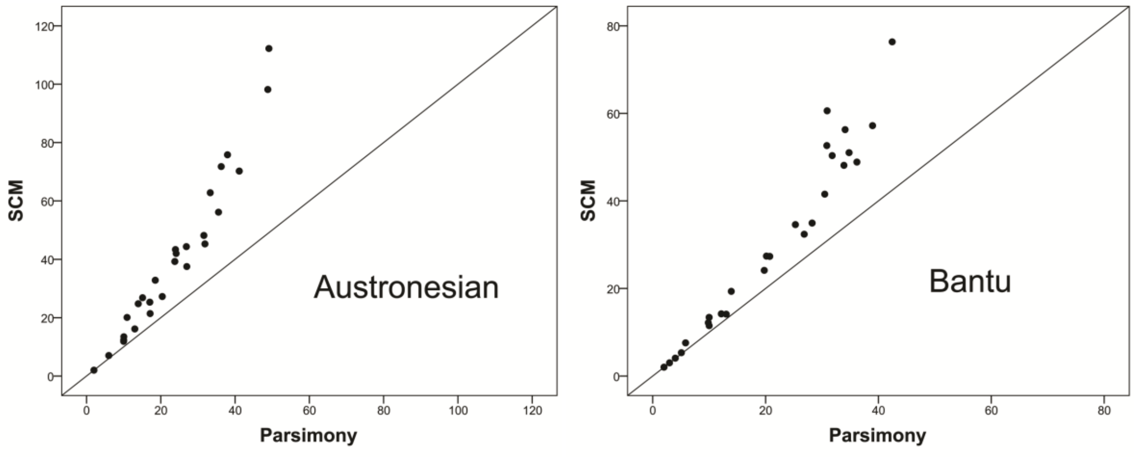

Figure S2. *Relative number of changes inferred under MP and SCM analyses are comparable for both Bantu and Austronesian. As SCM allows more than one change per branch, it always produces estimates that are equal to or higher than those under MP (the line indicates y=x)(NB: excludes v15).*

The ML analyses were conducted in the program BayesTraits (http://www.evolution.reading.ac.uk/BayesTraits.html) using the MULTISTATE algorithm and specifying a model of evolution in which all rate parameters take the same estimated value [10]. ML analyses of variable 38 (Segregation of Adolescent Boys) in Austronesian societies initially indicated a mean rate value of 2.73, which was an order of magnitude greater than any of the other rate estimates. Closer inspection of the output revealed that this inflated value was driven by the estimates from two of the trees in the posterior sample. These trees produced rate values of 136 and 115 while all other trees produced rate values around 0.22 (standard deviation = 0.006). Therefore for the ML analyses presented below the mean rate value for this variable was calculated excluding these two outlying values, giving a mean of 0.22. The value this variable takes is not crucial to the overall results; excluding this variable from the correlational analyses presented in Table S6 does not substantially affect the results (e.g. the correlation between Bantu and Austronesian ML values is 0.477 ($p=0.012$) including v38, and 0.495 ($p=0.01$) excluding it).

The tables below show the correlations between the inferred numbers and rates of change and the potentially confounding factors of number of taxa, and number of categories, for both the Austronesian and Bantu results. In Austronesian (Table S3) parsimony scores are highly correlated with SCM scores, but neither parsimony nor SCM scores show a significant correlation with ML values. In Bantu (Table S4) SCM and parsimony scores are highly correlated and show moderate correlations with the ML rates. However, by partialling out the effects of number of taxa and number of categories a trait takes ML rates and Parsimony scores are highly correlated in both Bantu ($r=0.91$, d.f.=23, $p<0.001$) and Austronesian ($r=0.83$, d.f.=24, $p<0.001$). These results indicate that Parsimony, SCM, and ML provide comparable estimates of the degree to which different traits have changed over the phylogenies.

Austronesian

	Parsimony	SCM	ML	Number of taxa	Number of categories for each trait in AN	Number of categories for each trait EA
Parsimony	.	.969 (<.001)	.009 (.964)	.445 (.018)	.781 (<.001)	.325 (.092)
SCM		.	.115 (.561)	.394 (.038)	.693 (<.001)	.259 (.183)
ML			.	-.297 (.125)	-.457 (.014)	-.696 (<.001)
Number of taxa				.	.258 (.184)	.048 (.807)
Number of categories for each trait in AN					.	.712 (<.001)

For all correlations df=28

Table S4. *Correlation coefficients and p-values (Pearson's r) between estimated number of changes under Parsimony and SCM methods, rate value from ML analyses, and three potential control variables (number of taxa in tree, number of variable categories in the Ethnographic Atlas, number of variable categories present in the Austronesian sample) for the Austronesian sample*

Bantu

	Parsimony	SCM	ML	Number of taxa for each trait	Number of categories for each trait in BN	Number of categories for each trait in EA
Parsimony		.972 (<.001)	.445 (0.02)	0.017 (0.930)	.654 (<.001)	.467 (0.012)
SCM			.531 (0.004)	-0.095 (0.636)	.639 (<.001)	.426 (0.027)
ML				-0.432 (0.024)	-0.179 (0.371)	-0.295 (0.135)
Number of taxa for each trait					0.022 (0.913)	0.1 (0.613)
Number of categories for each trait in BN						.823 (<.001)

For correlations involving ML and SCM df =27, otherwise df=28

Table S5. *Correlation coefficients and p-values (Pearson's r) between estimated number of changes under Parsimony and SCM methods, rate value from ML analyses, and three potential control variables (number of taxa in tree, number of variable categories in the Ethnographic Atlas, number of variable categories present in the Bantu sample) for the Bantu sample*

Control variables	Parsimony	SCM	ML
No control	0.65 (<.001)	0.592(0.001)	0.477(.012)
Number of taxa (AN &BN)	0.725(<.001)	0.669(<0.001)	0.357(.079)
Number of categories (EA)	0.596(.001)	0.552(0.003)	0.395(.046)
Number of categories (AN&BN)	0.328 (.102)	0.442 (0.027)	0.463(.02)
Number of taxa (AN &BN)+ Number of categories (EA)	0.670(<.001)	0.628(0.001)	0.230(.280)
Number of taxa (AN &BN)+ Number of categories (AN&BN)	0.428(0.037)	0.503(0.014)	0.304(.158)

Table S6. *Correlation coefficients (and p-values) of the associations between the estimates for Austronesian and Bantu under the three different methods.*

The main text presents the results from the correlations between parsimony scores for the Austronesian and the Bantu datasets. The following tables demonstrate that these overall findings are robust to the method used to estimate the amount or rate of character changes. Parsimony, SCM, and ML analyses all show significant positive correlations between the estimates from the Austronesian and the Bantu data. The correlations between estimated number of changes in Bantu and Austronesian datasets are robust to different combinations of control variables. The ML analyses still show a correlation when controlling for number of categories. This is not the case when controlling for the number of taxa, however, tables S3 and S4 show an inconsistent relationship between number of taxa and the ML rate value in Bantu and Austronesian (in Bantu there is a significant negative relationship, while in Austronesian there is no significant relationship). This suggests that number of taxa may not need to be controlled for in the ML analyses. More generally the results from the Parsimony and the SCM analyses show a consistent pattern, where as the results from the ML analyses are a little more complicated. This may be due to the difficulty of accurately estimating rate values in ML, particularly if the likelihood surface is quite flat, which will introduce error into the rate estimates. This error in estimating the rate values is likely to obscure any real relationship, rather than systematically introducing a bias in favour of finding a relationship that does not really exist. Furthermore, as we mentioned above the interpretation of the instantaneous rate parameters from ML is by no means straightforward. Interestingly, Dediu [11, 12] also compared the results of using different phylogenetic comparative methods in analyses of the stability of linguistic structural features. He used the evolutionary-model-based program BayesPhylogenies (which is usually used for inferring phylogenetic relationships) and the custom built program BayesLang (which employs a Bayesian algorithm for inferring the number of trait changes in a manner similar to maximum parsimony) and found that both methods gave broadly comparable results.

**Variable name**	**Bantu**			**Austronesian**		
	Parsimony	SCM	ML	Parsimony	SCM	ML
v6	*13.03*	*14.14*	*0.67*	*31.89*	*45.27*	*0.05*
v8	*38.96*	*57.19*	*2.78*	*49.11*	*112.22*	*0.12*
v9	*10.01*	*11.50*	*2.25*	*13.00*	*16.15*	*0.06*

v11	*19.77*	*24.14*	*2.31*	*37.96*	*75.81*	*0.18*
v15	*47.20*	*9230.15*	*503.20*	*33.37*	*52.19*	*0.08*
v24	*34.10*	*56.28*	*2.70*	*48.81*	*98.17*	*0.10*
v27	*20.16*	*27.40*	*1.52*	*35.54*	*56.15*	*0.07*
v29	*25.29*	*34.58*	*2.34*	*17.13*	*21.41*	*0.05*
v30	*36.19*	*48.88*	*1.67*	*27.00*	*37.52*	*0.06*
v32	*30.86*	*52.64*	*5.40*	*23.78*	*39.25*	*0.15*
v33	*42.44*	*76.33*	*3.95*	*33.32*	*62.80*	*0.12*
v34	*10.00*	*13.41*	*4.60*	*10.92*	*20.08*	*0.26*
v37	*13.93*	*19.34*	*4.12*	*15.10*	*26.84*	*0.28*
v38	*5.83*	*7.58*	*2.27*	*13.94*	*24.78*	*0.22*
v39	*2.98*	*3.02*	*0.63*	*10.05*	*13.48*	*0.10*
v40	*20.74*	*27.33*	*2.79*	*20.41*	*27.25*	*0.07*
v42	*4.00*	*4.08*	*0.39*	*10.01*	*12.31*	*0.09*
v43	*28.25*	*34.94*	*1.36*	*41.13*	*70.22*	*0.10*
v66	*34.80*	*51.03*	*2.82*	*36.27*	*71.77*	*0.14*
v70	*9.85*	*12.18*	*2.33*	*18.52*	*32.82*	*0.27*
v72	*30.49*	*41.54*	*1.76*	*31.60*	*48.17*	*0.06*
v76	*33.89*	*48.12*	*1.71*	*26.89*	*44.33*	*0.08*
v77	*31.81*	*50.36*	*3.56*	*17.04*	*25.31*	*0.09*
v79	*12.16*	*14.21*	*1.34*	*6.00*	*7.03*	*0.02*
v80	*2.00*	*2.03*	*0.20*	*23.95*	*43.34*	*0.20*
v81	*30.91*	*60.59*	*3.39*	*24.14*	*41.99*	*0.13*
v82	*26.85*	*32.39*	*1.06*	*10.00*	*11.89*	*0.03*
v83	*5.08*	*5.33*	*0.34*	*2.00*	*2.02*	*0.02*

Table S7. *Comparison of number of changes under parsimony and SCM analyses, and the value of the rate parameter from the ML analyses*

8. Evolutionary rates and phylogenetic signal

Some previous studies have examined rates of change in linguistic data partially with the aim of identifying features that could be used to examine deeper connections between languages and language families [11, 13]. We should stress that this is not our goal in this study. Indeed, the kind of ethnographic data used here is not well-suited to phylogenetic inference due to the fact that it is coded into a limited number of states, with a high possibility that distantly-related societies will converge on the same state. In this respect it is more similar to structural linguistic data rather than lexical data [11]. Furthermore, although we are using phylogenies to examine relative amounts of change in different we are not explicitly assessing the degree to which different traits are predicted by linguistic relationships, i.e. we are not assessing the "phylogenetic signal" in the data. This is an interesting issue and has been the focus of a number of studies [3, 14-18], however it is beyond the scope of the present study. As Revell and colleagues [19] point out there is a complicated relationship between evolutionary rates and processes on the one hand, and phylogenetic signal on the other.

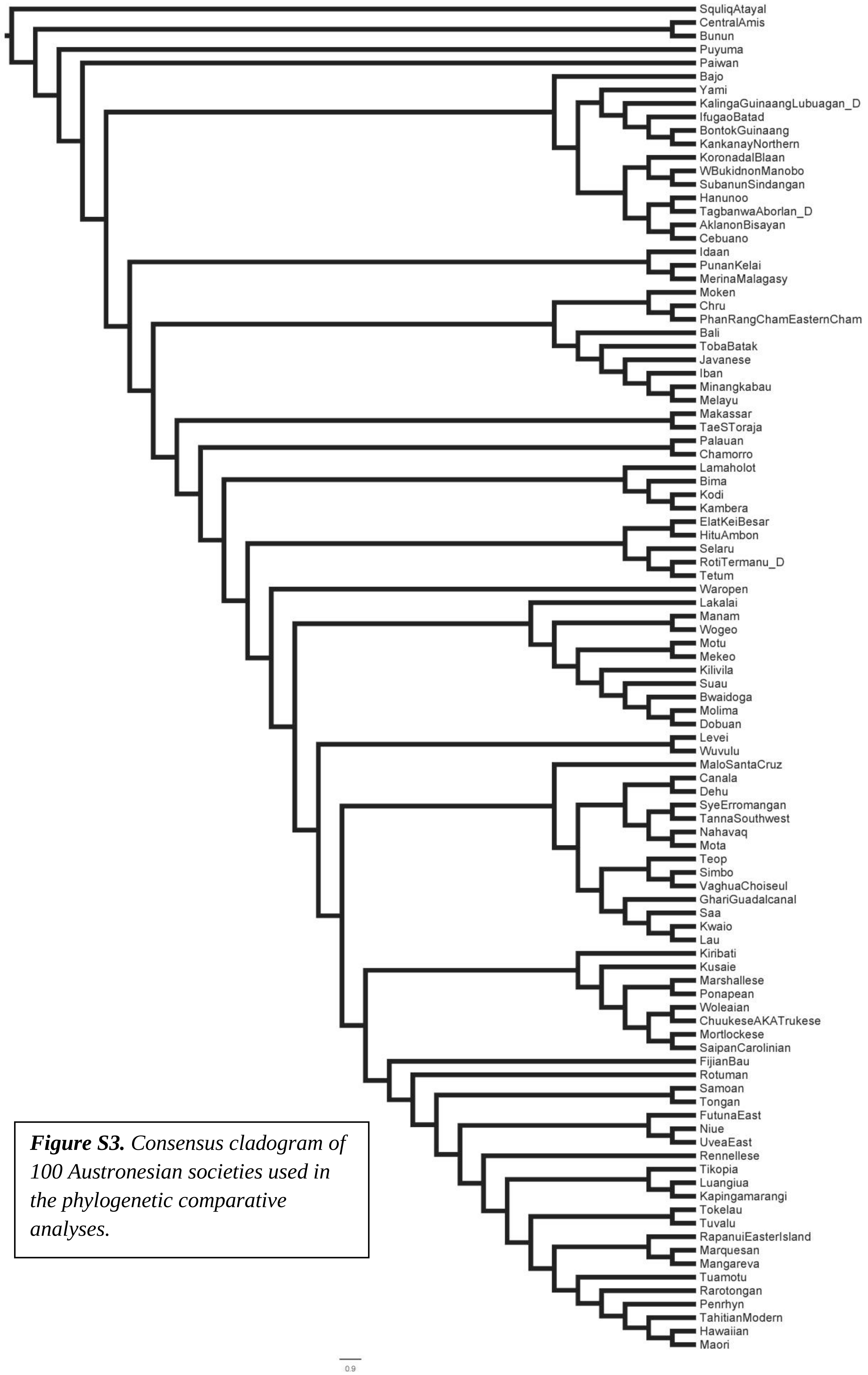

Figure S3. *Consensus cladogram of 100 Austronesian societies used in the phylogenetic comparative analyses.*

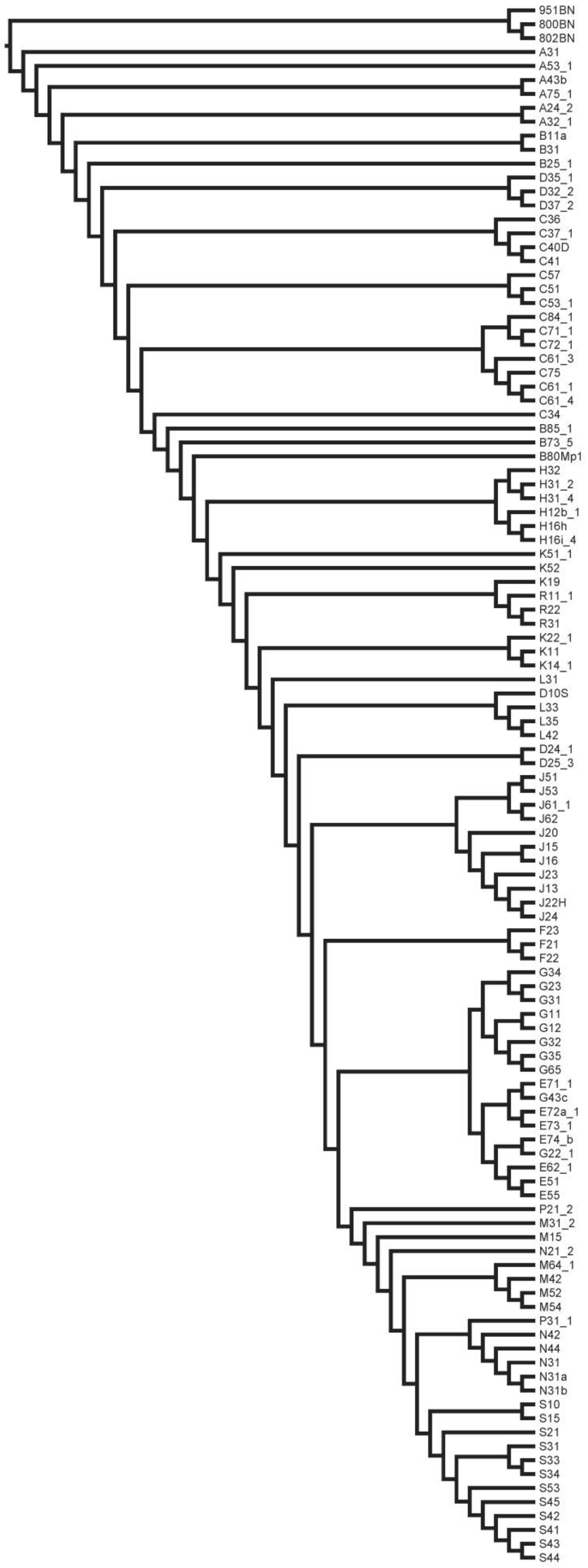

Figure S4. *Consensus cladogram of 112 Bantu societies used in the phylogenetic comparative analyses.*

Supplementary References

1. *Bastin Y., Coupez A., Mann M. 1999 Continuity and divergence in the Bantu languages: perspectives from a lexicostatic study. Tervuren, Belgium, Musée royal de l'Afrique centrale.*
2. *Greenhill S.J., Blust R., Gray R.D. 2008 The Austronesian Basic Vocabulary Database: From Bioinformatics to Lexomics. Evolutionary Bioinformatics, 271-283.*
3. *Guglielmino C.R., Viganotti C., Hewlett B., Cavallisforza L.L. 1995 Cultural Variation in Africa - Role of Mechanisms of Transmission and Adaptation. Proceedings of the National Academy of Sciences of the United States of America* ***92****(16), 7585-7589.*
4. *Dow M.M. 2007 Galton's Problem as Multiple Network Autocorrelation Effects: Cultural Trait Transmission and Ecological Constraint. Cross-Cultural Research* ***41****(4), 336-363. (doi:10.1177/1069397107305452).*
5. *Currie T.E., Meade A. 2014 Keeping Yourself Updated: Bayesian Approaches in Phylogenetic Comparative Methods with a Focus on Markov Chain Models of Discrete Character Evolution. In Modern Phylogenetic Comparative Methods and Their Application in Evolutionary Biology (ed. Garamszegi L.). Berlin Heidelberg, Springer-Verlag.*
6. *Fitzpatrick J.L., Montgomerie R., Desjardins J.K., Stiver K.A., Kolm N., Balshine S. 2009 Female promiscuity promotes the evolution of faster sperm in cichlid fishes. Proceedings of the National Academy of Sciences* ***106****(4), 1128-1132. (doi:10.1073/pnas.0809990106).*
7. *Currie T.E., Greenhill S.J., Gray R.D., Hasegawa T., Mace R. 2010 Rise and fall of political complexity in island South-East Asia and the Pacific. Nature* ***467****(7317), 801-804.*
8. *Mooers A.O., Schulter D. 1999 Reconstructing Ancestor States with Maximum Likelihood: Support for One-and Two-Rate Models. Systematic Biology.*
9. *Pagel M., Meade A. 2006 Bayesian analysis of correlated evolution of discrete characters by reversible-jump Markov chain …. Am Nat.*
10. *Pagel M., Meade A., Barker D. 2004 Bayesian estimation of ancestral character states on phylogenies. Systematic Biology* ***53****(5), 673-684.*
11. *Dediu D., Levinson S.C. 2012 Abstract Profiles of Structural Stability Point to Universal Tendencies, Family-Specific Factors, and Ancient Connections between Languages. PLoS ONE* ***7****(9), e45198.*
12. *Dediu D. 2011 A Bayesian phylogenetic approach to estimating the stability of linguistic features and the genetic biasing of tone. Proceedings of the Royal Society B: Biological Sciences* ***278****(1704), 474-479. (doi:10.1098/rspb.2010.1595).*
13. *Greenhill S.J., Atkinson Q.D., Meade A., Gray R.D. 2010 The shape and tempo of language evolution. Proceedings of the Royal Society B: Biological Sciences, -. (doi:10.1098/rspb.2010.0051).*
14. *Collard M., Shennan S.J., Tehrani J.J. 2006 Branching, blending, and the evolution of cultural similarities and differences among human populations. Evolution and Human Behavior* ***27****(3), 169-184.*
15. *Matthews L.J., Tehrani J.J., Jordan F.M., Collard M., Nunn C.L. 2011 Testing for Divergent Transmission Histories among Cultural Characters: A Study Using Bayesian Phylogenetic Methods and Iranian Tribal Textile Data. PLoS ONE* ***6****(4). (doi:e14810 10.1371/journal.pone.0014810).*
16. *Eff E.A. 2004 Does Mr. Galton still have a Problem? Autocorrelation in the Standard Cross-Cultural Sample. World Cultures* ***15****(2), 153-170.*
17. *Jordan P., Shennan S. 2005 Cultural Transmission in Indigenous California. In The Evolution of Cultural Diversity: A Phylogenetic Approach (eds. Mace R., Holden C.J., Shennan S.), pp. 165-196. London, Left Coast Press.*
18. *Mace R., Jordan F.M. 2011 Macro-evolutionary studies of cultural diversity: a review of empirical studies of cultural transmission and cultural adaptation. Philosophical Transactions of the Royal Society B: Biological Sciences* ***366****(1563), 402-411. (doi:10.1098/rstb.2010.0238).*
19. *Revell L.J., Harmon L.J., Collar D.C. 2008 Phylogenetic Signal, Evolutionary Process, and Rate. Systematic Biology* ***57****(4), 591-601. (doi:10.1080/10635150802302427).*
